# Whole Genome Sequencing of **Klebsiella pneumoniae** Strain Unravels a New Model for the Development of Extensive Drug Resistance in Enterobacteriaceae

**DOI:** 10.2174/1874285801812010195

**Published:** 2018-06-29

**Authors:** Mubarak Alfaresi

**Affiliations:** College of Medicine, University of Sharjah, Sharjah, UAE

**Keywords:** *Klebsiella pneumoniae*, Extensive drug resistance (XDR), Whole genome sequencing, Antibiotics, WHO, Enterobacteriaceae

## Abstract

**Introduction:**

Increased incidence of carbapenem-resistant Enterobacteriaceae (CRE) has been reported worldwide. The WHO warns about the imminent risk to global health if the spread of resistant bacteria is not contained.

**Materials and Methods:**

Here, single molecule real time sequencing was used to analyse the whole genome and resistome of SKGH01, a strain of **Klebsiella pneumoniae**.

**Results and Discussions:**

The data showed that SKGH01 was resistant to all commercially available antibiotics. A complete account of extensively drug-resistant (XDR) CRE at a genomic level and the entire location map of all antibiotic resistance components are here presented. Additionally, this work proposes a model of XDR acquisition in Enterobacteriaceae.

## 1 INTRODUCTION

**Klebsiella pneumoniae** of the Enterobacteriaceae family is a non-motile, rod-shaped, Gram-negative bacterium and it is one of the primary causes of hospital-acquired infections globally [1]. *K. pneumoniae* genomes have a strong virulence and a wide array of resistance factors that make them a major source of antimicrobial resistance genes [2]. The *K. pneumoniae* that produce carbapenemase (KPC-KP) are the most challenging pathogens. They exhibit extensive drug-resistant phenotypes and high potential for rapid spread having an overwhelming impact on morbidity and mortality rates [3]. Colistin and polymyxin B are antimicrobial agents that, for the most part, are still active against KPC-KP [4]. However, the emergence of polymyxin-resistant KPC-KP has recurrently been reported [5]. In *K. pneumoniae*, resistance to cationic antimicrobial agents is facilitated via lipopolysaccharide (LPS) sequence alterations driven by the pbgPE operon products, which are highly conserved among Enterobacteriaceae [6, 7]. The PhoQ/PhoP and PmrAB signalling systems positively regulate the pbgPE operon [7]. Activation of the PhoQ/PhoP signalling system induces production of a transmembrane regulatory protein called MgrB. The protein acts as a negative feedback loop on this signalling system by interacting with the PhoQ sensor kinase [8]. The MgrB protein has been shown to have this regulatory function in *Salmonella enterica, Escherichia coli* as well as *Yersinia pestis* and thus might also be conserved in other species, including *K. pneumoniae* [8].

## 2 MATERIAL AND METHODS

The Hospital Medical Executive Committee approved the study. The SKGH01 strain was isolated from an 80-year-old man with urinary tract infection. The species was characterised with the VITEK II compact GN system (bioM´erieux, France). For the antimicrobial susceptibility testing the VITEK II N211 system (bioM´erieux) and the E-test method were used. Breakpoints published by the Clinical and Laboratory Standards Institute were applied to determine the susceptibility to the tested antibiotics and the European Committee for Antibiotic Susceptibility Testing breakpoints in the E-test were used to determine the minimum inhibitory concentration of colistin. *K. pneumoniae* SKGH01 genome was sequenced with the Pacific Biosciences (PacBio, Inc., CA) RS II Single-Molecule Real Time (SMRT) kit. Bell template libraries were prepared using the Template Preparation Kit (PacBio). A single, streamlined protocol was used to create libraries of varying insert lengths, from 250 bp to 20,000 bp. The PacBio SMRT analysis software suite (v. 3.0) and hierarchical genome assembly process were used for *de novo* genome assembly. For the gene calling and automatic functional annotation of SKGH01 chromosome and plasmids the Prokka v1.12b (Vicbioinformatics, Australia) software was used. ResFinder and PlasmidFinder with data from the Center for Genomic Epidemiology (CGE) were employed to analyse the antimicrobial resistance genes and plasmid types. The Antibiotic Resistance Genes Database [9] and the Comprehensive Antimicrobial Resistance Database [10] were compared to all the predicted coding regions in order to screen the outstanding antimicrobial resistance genes. The insertion sequences (IS) in the genome were identified with the online tool, ISfinder 2 (version 2016-05-27). Closely related bacterial genomes were identified with the Microbial Nucleotide BLAST program. The search set consisted of complete genomes of *K. pneumoniae* (taxid: 573) available in the NCBI database. The BLAST search produced 48 significant hits, with overall similarities between 95% and 99%, and coverages between 85% and 98%. A genome tree was built, which comprised SKGH01 and 40 related strains from NCBI database (accession date: 10/05/16).

## 3 RESULTS AND DISCUSSION

The data showed that SKGH01 is a true extensively Drug-Resistant (XDR) strain to ampicillin, ampicillin-clavulanic acid, piperacillin-tazobactam, cefotaxime, ceftazidime, cefepime, aztreonam, meropenem, cotrimoxazole, amikacin, gentamicin, and colistin. A total of 6 contigs representing 6,088,457 bases (GC content 56.54%, N50=10,230) were obtained from assembled sequences of strain SKGH01 (Table **S1**). 6,034 genes (total), 5,907 CDS (total), 5,777 genes (coding), and 127 tRNAs genes were annotated for final contigs. The complete genome of K. pneumoniae SKGH01 consists of a circular chromosome 5,490,611 base-pairs in length with an average G-C content of 56.4%, four circular plasmids. The complete genome of strain SKGH01 consisted of a circular chromosome (5,490,611 base-pairs long) with an average G-C content of 56.4%, and four circular plasmids. Most of the genes for acquired resistance to antibiotics were positioned on the chromosome. The complete resistomes of strain SKGH01 are presented in Table **1**. The insertion sequence, ISEcp1 (synonym, ISEc9) was found in four and blaOXA-181 in three places on the SKGH01 chromosome. The search for the (partial) protein sequence encoded by mgrB was performed. The most significant tblast match was a 42-amino acid, 5’ partial sequence of mgrB, which corresponded to the first ISEcp1 position identified on the SKGH01 chromosome. The remaining 3’ partial sequence of mgrB was identified with a manual search. Another manual search identified left- and right-flanking, inverted repeats (IRL and IRR, respectively) located at the first ISEcp1 position on the chromosome. We also found two alternative IRRs (IRRalts), which produced the insertions ISEcp1-blaOXA-181-IRRalt1 and ISEcp1-blaOXA-181-IRRalt2. One of these insertions led to the inactivation of the mgrB gene (Fig. **S1**). ISEcp1-like insertion sequences are the most common genetic element associated with blaCTX-M, blaCMY and blaACC genes and have more recently been associated with blaOXA-181 [11].

## CONCLUSION

Here, using the long-read sequencing technology multiple, identical, carbapenem-resistance elements in the *K. pneumoniae* strain SKGH01 genome were identified. Based on the data, a new model explaining how XDR in this *K. pneumoniae* isolate developed via colistin resistance by mgrB gene disruption by ISEcp1. In this model, new resistance was driven by the existing mobile resistance determinants. Additionally, the data showed that ISEcp1 sequence interrupted the negative feedback regulator of the PhoQ-PhoP signalling system, namely the *mgrB* gene. Interestingly, this disruption was previously shown to drive the KPC-KPs acquired colistin resistance. Indeed, interruption of the *mgrB* gene caused upregulation of PhoQ-PhoP signalling; in turn, this upregulation activated the Pmr system, which was responsible for modifying the LPS target of polymyxin [12].

## Figures and Tables

**Table 1:** Resistome analysis for the SKGH01 strain of *K. pneumoniae.*

**START**	**STOP**	**Gene**	Identity %*	**Associated Resistance**
2637986	2638846	shv-11	100	beta-lactam resistance gene
1544531	1545253	baeR	91	aminocoumarin resistance gene; aminoglycoside resistance gene;
2009887	2010294	h-ns	94	macrolide resistance gene; fluoroquinolone resistance gene; tetracycline resistance gene; beta-lactam resistance gene
253419	254558	acrE	75	beta-lactam resistance gene; fluoroquinolone resistance gene
489403	490224	bacA	89	peptide antibiotic resistance gene
1546725	1548140	mdtD	84	efflux pump conferring antibiotic resistance
95184	95666	dfrA14	99	trimethoprim resistance gene
201118	201750	crp	99	macrolide resistance gene; beta-lactam resistance gene; fluoroquinolone resistance gene
59699	60490	aadA25	99	antibiotic inactivation enzyme; aminoglycoside resistance gene
98949	99593	qnrB1	100	antibiotic target protection protein; fluoroquinolone resistance gene
2392402	2392854	arr-2	100	rifampin resistance gene
3410534	3411766	mdfA	85	efflux pump conferring antibiotic resistance
4527671	4528912	mdtM	75	efflux pump conferring antibiotic resistance
2389793	2390347	aac(6')-Ib9	99	aminoglycoside resistance gene
4436582	4437451	robA	82	chloramphenicol resistance gene; fluoroquinolone resistance gene; tetracycline resistance gene; rifampin resistance gene; beta-lactam resistance gene
1856894	1857691	oxa-181	100	beta-lactam resistance gene
3487775	3489415	pmrC	70	polymyxin resistance gene
53618	54256	cat	100	chloramphenicol resistance gene
945981	947153	emrA	81	efflux pump conferring antibiotic resistance; fluoroquinolone resistance gene
86466	88451	arnA	77	polymyxin resistance gene
1190757	1193870	mexD	91	chloramphenicol resistance gene; macrolide resistance gene; fluoroquinolone resistance gene
514487	516745	parC	94	fluoroquinolone resistance gene
5231835	5233013	mdtL	75	efflux pump conferring antibiotic resistance
4048769	4049566	oxa-181	100	beta-lactam resistance gene
250296	253406	mexD	86	chloramphenicol resistance gene; macrolide resistance gene; fluoroquinolone resistance gene
1554342	1555580	mdtA	79	aminocoumarin resistance gene
3489415	3490086	pmrA	78	polymyxin resistance gene
947279	947809	emrR	92	fluoroquinolone resistance gene;
2617505	2617882	marA	92	chloramphenicol resistance gene; fluoroquinolone resistance gene; tetracycline resistance gene; rifampin resistance gene; beta-lactam resistance gene
5125322	5126695	cpxA	94	aminocoumarin resistance gene; aminoglycoside resistance gene
3123628	3124299	phoP	91	polymyxin resistance gene; macrolide resistance gene
944427	945965	emrY	94	efflux pump conferring antibiotic resistance; tetracycline resistance gene
2219510	2220883	mdtK	87	fluoroquinolone resistance gene
5124627	5125325	cpxR	94	efflux pump conferring antibiotic resistance; aminocoumarin resistance gene; aminoglycoside resistance gene; gene modulating antibiotic efflux
3892696	3893781	acrA	86	chloramphenicol resistance gene; fluoroquinolone resistance gene; efflux pump conferring antibiotic resistance; tetracycline resistance gene; rifampin resistance gene; beta-lactam resistance gene
3308977	3309852	ctx-M-15	100	antibiotic inactivation enzyme; beta-lactam resistance gene
5075084	5075881	oxa-181	100	beta-lactam resistance gene
5278571	5279755	emrD	99	efflux pump conferring antibiotic resistance
4543523	4543945	fosA5	97	fosfomycin resistance gene
2388603	2389382	rmtF	100	aminoglycoside resistance gene
85486	86469	pmrF	83	polymyxin resistance gene; gene altering cell wall charge conferring antibiotic resistance
3757172	3757513	ramA	92	chloramphenicol resistance gene; fluoroquinolone resistance gene; tetracycline resistance gene; rifampin resistance gene; beta-lactam resistance gene
1412712	1415345	gyrA	92	fluoroquinolone resistance gene
3875438	3877114	rosB	73	polymyxin resistance gene
60898	61395	dfrA12	100	antibiotic target replacement protein; trimethoprim resistance gene
3190885	3192093	mdtH	85	efflux pump conferring antibiotic resistance
2386317	2386955	cat	100	chloramphenicol resistance gene
3124299	3125765	phoQ	81	polymyxin resistance gene; macrolide resistance gene
57095	57649	aac(6')-Ib9	99	antibiotic inactivation enzyme; aminoglycoside resistance gene
503638	505116	tolC	83	chloramphenicol resistance gene; macrolide resistance gene; fluoroquinolone resistance gene; aminocoumarin resistance gene; tetracycline resistance gene; rifampin resistance gene; beta-lactam resistance gene
3198868	3199821	mdtG	84	efflux pump conferring antibiotic resistance
1598283	1599449	pmrE	82	polymyxin resistance gene
3893804	3896950	mexD	92	efflux pump conferring antibiotic resistance; chloramphenicol resistance gene; macrolide resistance gene; fluoroquinolone resistance gene
55905	56684	rmtF	100	aminoglycoside resistance gene
1545250	1546728	baeS	77	aminocoumarin resistance gene; aminoglycoside resistance gene
987867	991019	oqxB	98.5	Quinolone resistance
991043	992218	oqxA	99.5	Quinolone resistance

* Percentage given by the Antibiotic Resistance Genes Database (ARDB) and the Comprehensive Antimicrobial Resistance Database (CARD) when compared with known resistance genes database.

## NUCLEOTIDE SEQUENCE ACCESSION NUMBER

The nucleotide sequence data are available in the GenBank nucleotide database, under accession numbers CP015500.1 to CP015505.1.
