# Supplementary material for: Whole Genome Sequencing of Klebsiella pneumoniae Strain Unravels a New Model for the Development of Extensive Drug Resistance in Enterobacteriaceae
Source: Open Microbiol J. 2018 Jun 29;12:195–9. doi: 10.2174/1874285801812010195 (PMC6047195; doi:10.2174/1874285801812010195)
Supplement: Supplementary file 1 [file TOMICROJ-12-195_SD1.pdf]

Send Orders for Reprints to reprints@benthamscience.ae

The Open Microbiology Journal, 2018, *12*, i

BENTHAM OPEN

CrossMark

The Open Microbiology Journal

Supplementary Material

Content list available at: www.benthamopen.com/TOMICROJ/

DOI: 10.2174/1874285801812010195

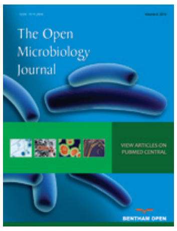

Supplementary Material

Whole Genome Sequencing of *Klebsiella Pneumoniae* Strain Unravels a New Model for the Development of Extensive Drug Resistance in Enterobacteriaceae

Mubarak Alfaresi*

College of Medicine, University of Sharjah, Sharjah, UAE

Received: March 15, 2018 Revised: June 5, 2018 Accepted: June 6, 2018

SUPPLEMENTARY FIGURE AND TABLE

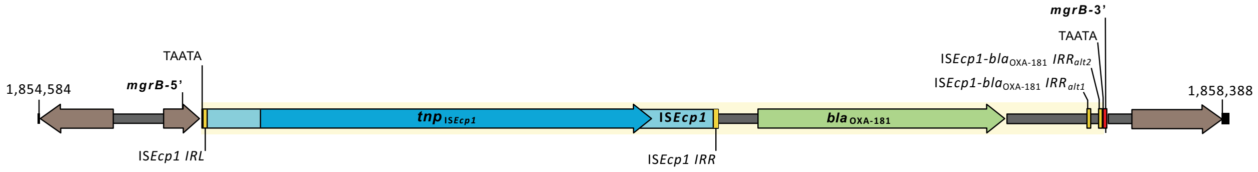

Fig. (S1). mgrB gene disruption by ISEcp1. The ISEcp1 element is represented as blue rectangle. Protein-coding genes are represented by colored arrows: blaOXA-181 (green), ISEcp1 transposase (dark blue), mgrB 5' and 3' parts (red), other (brown). Left and right flanking inverted repeats (IRL, IRR, IRRalt1, IRRalt2) are represented by yellow bars and 5-bp direct repeat sequences are given (TAATA). Yellow shading indicates the sequence region inserted into mgrB.

Table S1. Associations between gene polymorphisms and their related proteins.

Contig Name	Length(bp)	GC%	Depth	Alias
**Contig1**	**5,490,611**	**57.3**	**128**	**Contig1**
**Contig2**	**281,190**	**46.8**	**219**	**Contig2**
**Contig3**	**140,874**	**52.1**	**208**	**Contig3**
**Contig4**	**84,941**	**52.7**	**295**	**Contig4**
**Contig5**	**56,230**	**53**	**350**	**Contig5**
**Contig6**	**34,611**	**48.6**	**181**	**Contig6**
**Total**	**6,088,457**			

© 2018 Mubarak Alfaresi.

This is an open access article distributed under the terms of the Creative Commons Attribution 4.0 International Public License (CC-BY 4.0), a copy of which is available at: (https://creativecommons.org/licenses/by/4.0/legalcode). This license permits unrestricted use, distribution, and reproduction in any medium, provided the original author and source are credited.

1874-2858/18 2018 Bentham Open
